# A DPO-Enhanced Gold Nanoparticle-Assisted PCR Assay for Simultaneous Detection of Bovine Viral Diarrhea Virus, Astrovirus, and Rotavirus

**DOI:** 10.3390/ani16060914

**Published:** 2026-03-14

**Authors:** Dongjie Cai, Yulin Lin, Jie Chen, Bin Tian, Qing Liu, Xiaoping Ma, Jiabin Gao, Zhicai Zuo

**Affiliations:** 1 Key Laboratory of Agricultural Animal Diseases and Veterinary Public Health of Sichuan Province, College of Veterinary Medicine, Sichuan Agricultural University, Chengdu 611130, China; dongjie_cai@sicau.edu.cn (D.C.);; 2 Xuanhan County Animal Disease Prevention and Control Center, Dazhou 636150, China; 3 Institute of Preventive Veterinary Medicine, Sichuan Agricultural University, Chengdu 611130, China; 4 Baicheng Institute of Animal Husbandry, Baicheng 137000, China

**Keywords:** dual-priming oligonucleotide, nanoparticle-assisted PCR assay, bovine viral diarrhea virus, bovine rotavirus, bovine astrovirus

## Abstract

To develop a rapid detection tool for major bovine diarrheal viruses, Bovine viral diarrhea virus (BVDV), Bovine astrovirus (BAstV), and Bovine rotavirus (BRV), a gold nanoparticle-enhanced dual-priming oligonucleotide (DPO) multiplex PCR method was established. After optimization, the assay showed high sensitivity with detection limits of 4.9 × 10^−1^, 2.72 × 10^2^, and 1.88 × 10^3^ copies/μL for BVDV, BAstV, and BRV, respectively, and demonstrated excellent specificity. In the validation using 963 clinical samples, the DPO-nanoPCR showed numerically higher detection rates for BVDV (5.50% vs. 4.36%) and BAstV (1.04% vs. 0.62%) compared to conventional PCR, while the detection rate for BRV was identical (0.93%). Statistical analysis indicated no significant difference between the two methods (*p* > 0.05), with an overall agreement rate exceeding 98.86%. The developed DPO-nanoPCR method provides a sensitive, specific, and efficient tool for the simultaneous surveillance and diagnosis of these key bovine enteric pathogens.

## Simple Summary

Rapid detection of the viruses causing diarrhea in cattle is crucial for safeguarding herd health, as existing tests sometimes lack efficiency and speed. The present work devised a swift, integrated diagnostic approach capable of concurrently identifying the three primary viruses responsible for diarrhea in cattle. The methodology integrated specifically engineered primers with nanoparticles, yielding a very sensitive assay capable of detecting extremely low viral concentrations. This novel method identified a greater number of illnesses than the traditional test when applied to numerous field samples, while exhibiting dependable and consistent outcomes. This effective technology provides farmers and veterinarians with a practical method for fast identification and monitoring of these viruses, facilitating prompt action to protect herd health and prevent significant economic losses.

## 1. Introduction

Bovine Viral Diarrhea Virus (BVDV), Bovine Astrovirus (BAstV), and Bovine Rotavirus (BRV) are the primary pathogens that induce both singular and co-infections in calves exhibiting diarrhea symptoms. These viruses represent a substantial danger to the economic sustainability and animal health within the worldwide cattle industry [1,2,3]. BVDV infection causes diarrhea and reproductive abnormalities in cattle and can lead to persistent infections, wherein sick calves repeatedly shed the virus into the environment, serving as a key transmission source to other cattle [4,5]. BRV, a principal etiological agent of viral diarrhea in calves, demonstrates significant infectivity and pathogenicity, frequently resulting in severe dehydration and, in certain instances, death [6,7]. BAstV infection induces pathological changes, including necrosis and desquamation of ileal epithelial cells in cattle. Furthermore, co-infection with additional intestinal pathogens, such as BVDV and BRV, markedly intensifies the severity of diarrhea symptoms [2]. Consequently, the development of rapid and accurate detection techniques is fundamental for effective prevention and control strategies. Nevertheless, owing to the genetic variety of these three viruses and the significant changes in their epidemiological and clinical characteristics across various areas and people, there is still a lack of technologies capable of concurrently detecting all three diseases.

Traditionally, laboratory diagnosis of these pathogens has mainly relied on virus isolation, enzyme-linked immunosorbent assay (ELISA), or singleplex PCR. However, these methods are hampered by limitations such as long detection periods, low throughput, high costs, and the inability to achieve simultaneous detection of multiple pathogens, thereby restricting large-scale clinical screening and rapid diagnosis of mixed infections [8,9,10]. In addition, techniques such as nucleic acid hybridization, RT-PCR, quantitative RT-PCR (qRT-PCR), CRISPR/Cas12a, and RT-LAMP have been widely used for viral nucleic acid detection. However, these methods typically require specialized equipment, skilled technicians, and well-equipped laboratories, limiting their application in field settings such as basic-level farms [11,12,13]. Meanwhile, although virus neutralization tests (VNT) and genome sequencing enable accurate genotyping, their high cost, complex operation, and strict requirements for laboratory platforms and bioinformatics analysis render them unsuitable for routine field diagnosis [14]. Multiplex PCR has developed as a potent method to simultaneously detect numerous diseases, offering significant improvements in efficiency, sensitivity, and cost-effectiveness [15,16,17]. Notwithstanding these advantages, conventional multiplex PCR is susceptible to primer cross-reactions and non-specific amplifications, especially when simultaneously detecting viruses with significant genomic discrepancies, complicating the equilibrium between sensitivity and specificity [18]. To address this issue, dual priming oligonucleotide (DPO) primer technology has been developed. By incorporating a spacer design, DPO primers can effectively mitigate non-specific binding, significantly enhancing the specificity and compatibility of multiplex PCR [19]. Additionally, DPO primers expand the range of suitable annealing temperatures, enhancing the robustness of the PCR analysis [20]. The integration of gold nanoparticles (GNPs) further augments the amplification efficiency of the DPO-PCR assay. GNPs enable the PCR system to reach the target temperature more rapidly and facilitate the generation of a greater number of amplicons through efficient heat transfer, thereby optimizing the reaction’s amplification efficiency [21]. As a result, DPO primers are particularly well-suited for the development of multiplex PCR assays [22,23,24]. Previously, Wang et al. (2019) developed a multiplex assay targeting several bovine enteric pathogens using this technology, demonstrating its preliminary utility in processing complex clinical samples [7].

In the present study, we developed a multiplex DPO-nanoPCR assay integrating DPO primers with nanotechnology, achieving the first simultaneous and highly efficient detection of BVDV, BAstV, and BRV. The performance of this assay, in terms of sensitivity, specificity, and reproducibility, was systematically evaluated by optimizing primer design, reaction systems, and amplification conditions. In addition, its practical applicability in clinical samples was assessed. The developed method exhibited higher sensitivity for BVDV and BAstV than conventional PCR approaches. Furthermore, extensive testing of clinical samples has demonstrated that this method is superior to other methods for detecting low viral loads in cattle population screening. The findings of this study offer crucial technical support for the swift diagnosis and epidemiological monitoring of bovine viral diarrhea syndrome, while also serving as a pertinent reference for the advancement of simultaneous detection methods for other co-infectious animal pathogens, highlighting its substantial scientific and practical significance.

## 2. Materials and Methods

### 2.1. Multiplex PCR Primer Design and Preparation of Recombinant Plasmids

Genes 5′UTR from BVDV, *ORF 1ab* from BAstV and *VP6* from BRV are highly conserved sequences and are commonly used in designing primers to specifically recognize viruses [25,26,27].The gene sequences of BVDV *5′-UTR* (classical isolate 103), BAstV *ORF 1ab* (classical strain HT4-TUR), and BRV *VP6* (classical strain ZAF/1162/2012) have been deposited in the GeneBank database (GenBank accession numbers for 5′UTR, *ORF 1ab*, and *VP6* are MW057258, MG957150, and MW771109, respectively), the most conserved nucleotide sequences were identified through comparison using DNA Star 7.1. Three pairs of standard conventional primers and DPO primers were designed using Oligo 6.0 and Primer 5 software, and were synthesized by Sangon Biotech (Shanghai, China). DPO primer involves inserting a polyhypoxanthine (poly I) linker into conventional primers. DPO primer design was adhered to the principles of DPO primer design: a 5′-terminal length of 18–25 bp with a melting temperature (Tm) > 65.0 °C, a 3′-terminal length of 6–12 bp, and a guanine-cytosine (G + C) mole fraction of 40–80%. Three viral plasmids with Amp^+^ resistance, constructed in the vector pUC57, were synthesized by Tsingke Biotech (Beijing, China) through their professional synthesis service. The information of primers and recombinant plasmids is shown in Table 1 and Table 2. The obtained bacterial cultures were subjected to plasmid extraction using the Mekhi Bio Plasmid Extraction Kit and subsequently stored at −20 °C.

### 2.2. Sample Collection and RNA/DNA Isolation

A total of 963 samples were collected, including 109 fecal samples from cattle across various beef cattle farms in Sichuan Province, 442 serum samples, and 412 anal swab samples (totaling 854) in the Ganzi region of Sichuan Province. The detailed sampling information is presented in Supplementary Table S1. Fecal and anal swab samples were immersed in phosphate-buffered saline (PBS), while serum samples were sealed. All samples were placed on ice within a foam box and transported back to the laboratory for further processing. For each collected anal swab or fecal sample, 1 mL of PBS buffer was added, followed by vortex-shaking for 15 s to ensure thorough mixing, generating the clinical samples for examination.

The viral DNA/RNA extraction kit from Accurate Biology (Changsha, China) was utilized to extract RNA from the samples. Subsequently, the extracted sample RNA was reverse-transcribed into complementary DNA (cDNA) using the reverse transcription kit from Lanbolide BioTech Co. (Beijing, China). The RNA extraction procedure was carried out strictly according to the kit manual. The reverse transcription procedure and the composition of the reaction system were as follows: 10 μL of template RNA, 4 μL of All-in-One First-Stand Synthesis Master Mix, 1 μL of dsDNase, and the volume was adjusted to 20 μL with Nuclease-Free Water. The reaction mixture was incubated at 37.0 °C for 2 min to eliminate genomic DNA contamination, then at 55.0 °C for 15 min, and finally terminated by incubation at 85.0 °C for 5 min. The resulting cDNA was promptly stored at −20.0 °C for subsequent experiments.

### 2.3. Preparation and Characterization of Gold Nanoparticles (GNPs)

Gold nanoparticles with an average diameter of 10 nm were synthesized using the Turkevich and Frens synthesis method [28]. Briefly, 100 mL of 0.01% gold chloride solution was boiled for 3 min. Then, 5 mL of pre-heated (37 °C) 1% trisodium citrate solution was rapidly added while stirring with a glass rod. The solution was boiled again for 5–8 min until it turned wine-red. After cooling at room temperature, the GNPs were visualized using a transmission electron microscope and stored at 4 °C. A small aliquot of the GNPs was protected from light, dispensed, and sent to Servicebio company (Wuhan, China) for electron microscopy analysis.

### 2.4. Optimization of DPO-nanoPCR Assay Conditions

The annealing temperature, primer concentration, and GNP concentration of the DPO-nanoPCR were optimized using the recombinant plasmid as a template. To assess the impact of different annealing temperatures on detection, reactions were carried out at temperatures ranging from 45 to 72 °C. Given the limited reagents in the multiplex PCR amplification system, where each primer pair competes for reaction components, the volumes and ratios of the three primer pairs were optimized. The volumes of all primers (10 mM) were adjusted from 0.1 to 1.0 mL in 0.1 mL increments. To evaluate the effect of GNPs on amplification, the volumes of GNPs (10 nM) were varied from 0.1 to 1.0 mL in 0.1 mL increments. A no-template control (NTC) was included in all experiments throughout this study to monitor potential contamination.

### 2.5. Sensitivity Analysis of DPO-nanoPCR

The positive plasmid standard template at a concentration of 1 ng/μL was diluted into 10 concentration gradients. PCR amplifications were then performed under the optimized conditions determined in the previous tests to evaluate the sensitivity of the PCR methods. All PCR amplifications were set with three technical replicates.

Plasmid copy number (copies/µL) was calculated according to the following equation: [6.02 × 10^23^ (copy/mol) × DNA amount (g) × 10^−9^)/(DNA length (bp) × 660 (g/mol/bp)] [29].

### 2.6. Specificity Analysis of DPO-nanoPCR

For the multiplex PCR, amplification was performed using a mixed template of BVDV clinical isolate cDNA, BAstV, and BRV positive plasmid standards with multiplex primers. Additionally, the cDNAs of BCoV, BPIV3, IBRV, APPV, and PEDV viruses were used as templates with multiplex primers, respectively.

The specificity of the DPO-nanoPCR method developed in this study was examined by separately amplifying the cDNAs of BCoV, BPIV3, IBRV, APPV, and PEDV viruses as templates. Three technical replicates were performed.

### 2.7. Reproducibility Analysis of DPO-nanoPCR

To assess the intra-group reproducibility and stability of the PCR methods established in this experiment, the positive plasmid was used as a template. Amplifications were conducted at two-month intervals using different brands of 2× Premix Taq, under the optimized reaction system and conditions. The stability was further tested after six months of freezing storage. The experiment was performed in triplicate.

### 2.8. Detection of Clinical Samples

A total of 963 clinical samples were tested by the conventional PCR constructed in our lab and DPO-nanoPCR developed in this study. These results were compared with those from the conventional PCR assay using 2% agarose gel electrophoresis.

### 2.9. Statistical Analysis

The detection results were processed using SPSS 26 software, and χ^2^ comparison test was used for data analysis. A *p* value < 0.05 was considered statistically significant. Concordance rate = (Number of identical positives detected by both the conventional PCR method and the DPO-nanoPCR method + Number of identical negatives detected)/Total number of samples tested.

## 3. Results

### 3.1. Characterization of the Synthesized Gold Nanoparticles

**Figure 1:**
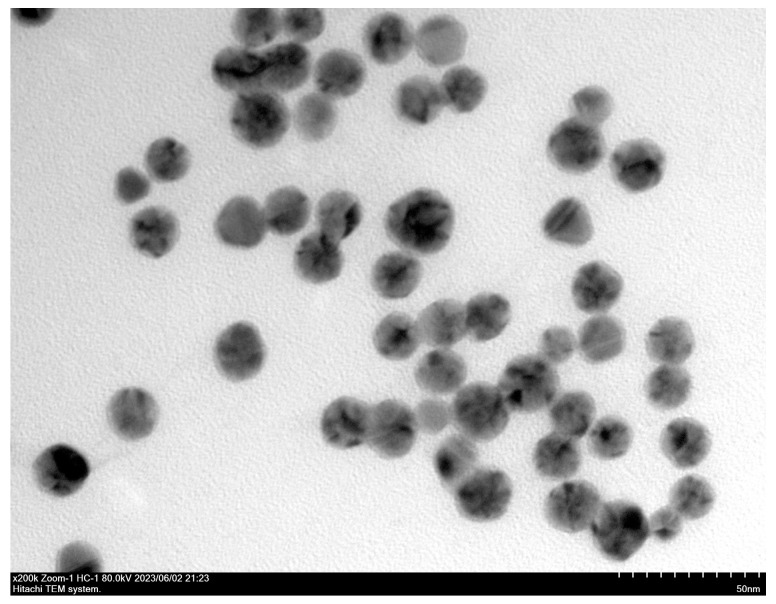
Transmission electron microscopy (TEM) image of synthesized gold nanoparticles (GNPs).

The synthesized GNPs exhibited a dark burgundy color, characterized by uniformity, transparency, and clarity. Under the transmission electron microscope, the GNPs demonstrated relatively regular spherical morphology and size, with particle sizes ranging from approximately 10 nm to 20 nm. They were uniformly dispersed, free of impurity fragments and agglomeration, as depicted in Figure 1. These morphological features suggest the successful synthesis of high-quality GNPs suitable for subsequent applications in the DPO-nanoPCR assay.

### 3.2. Optimizing the DPO-nanoPCR Assay

**Figure 2:**
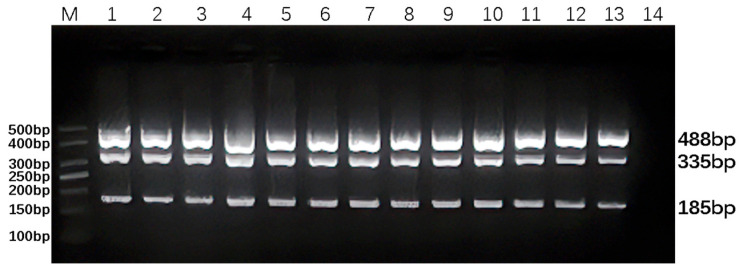
Optimization of the annealing temperature for DPO-nanoPCR. Lane M represents a 50 bp DNA Ladder Marker for size reference. Lanes 1–13 correspond to annealing temperatures of 45.0 °C, 47.0 °C, 49.0 °C, 51.0 °C, 53.0 °C, 55.0 °C, 57.0 °C, 59.0 °C, 61.0 °C, 63.0 °C, 65.0 °C, 67.0 °C, and 69.0 °C, respectively. Lane 14 serves as a negative control.

The annealing temperature of the DPO-nanoPCR for detecting BVDV, BAstV, and BRV, three common bovine diarrhea pathogens, was optimized within the temperature range of 45.0–69.0 °C. The results indicated that efficient amplification of BVDV, BAstV, and BRV by DPO-nanoPCR could be achieved within this temperature interval, as shown in Figure 2. Among these, 55.0 °C was determined as the optimal annealing temperature, which provided the best balance for specific and efficient amplification of the target viruses.

**Figure 3:**
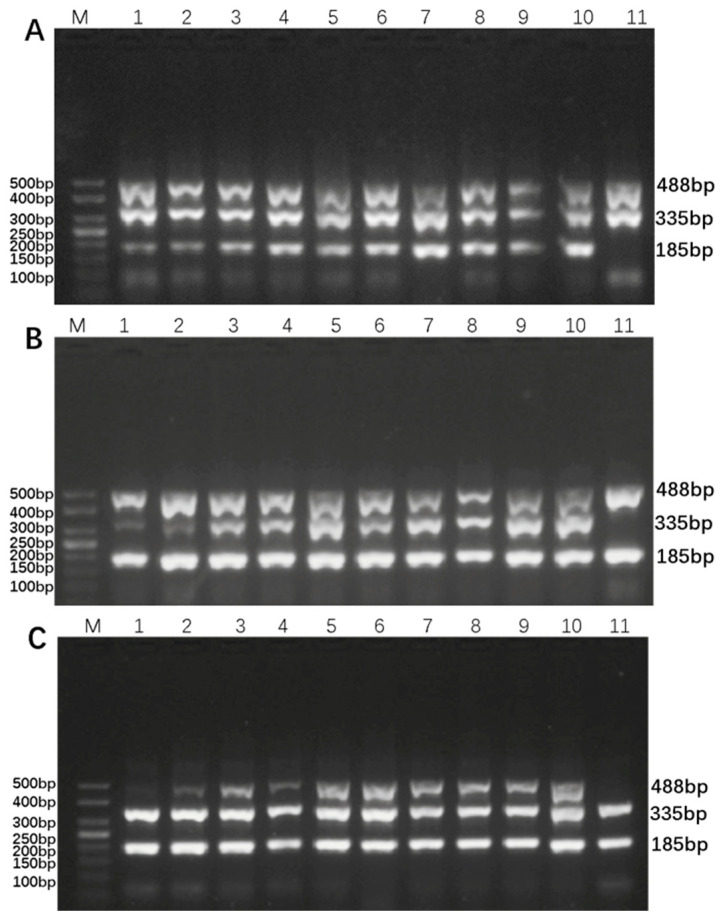
Optimization of primer dosage for DPO-nanoPCR. Lane M shows a 50 bp DNA Ladder Marker for size comparison. Panel (**A**) presents the optimization results for BVDV-specific DPO primers, panel (**B**) for BAstV-specific DPO primers, and panel (**C**) for BRV-specific DPO primers. Lanes 1–10 in each panel represent primer dosages of 0.1 μL, 0.2 μL, 0.3 μL, 0.4 μL, 0.5 μL, 0.6 μL, 0.7 μL, 0.8 μL, 0.9 μL, and 1.0 μL, respectively. Lane 11 in each panel is a negative control.

**Figure 4:**
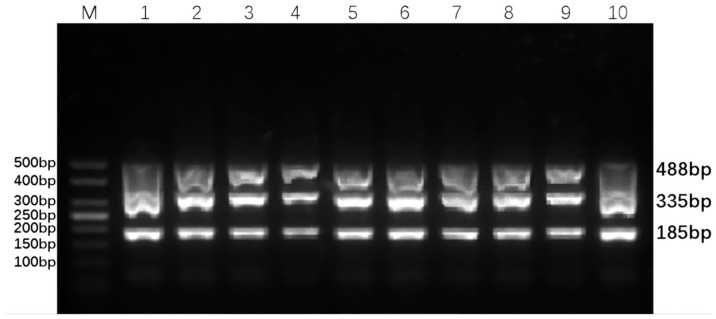
Optimization of gold nanoparticle (GNP) dosage for DPO-nanoPCR. Lane M is a 50 bp DNA Ladder Marker for sizing the PCR amplicons. Lanes 1–10 represent GNP dosages of 0.1 µL, 0.2 µL, 0.3 µL, 0.4 µL, 0.5 µL, 0.6 µL, 0.7 µL, 0.8 µL, 0.9 µL, and 1.0 µL, respectively.

Based on the electrophoresis results, the optimal primer amounts for maximum amplification efficiency were identified: 0.4 µL of each forward and reverse BVDV-DPO primer, 0.3 µL of the BAstV-DPO primer, and 0.3 µL of the BRV-DPO primer, as illustrated in Figure 3. Additionally, the DPO-nanoPCR amplification bands were found to be the clearest and brightest when the GNP dosage was 0.9 µL, as shown in Figure 4. These optimized conditions form the foundation for reliable and sensitive detection of the target viruses.

**Figure 5:**
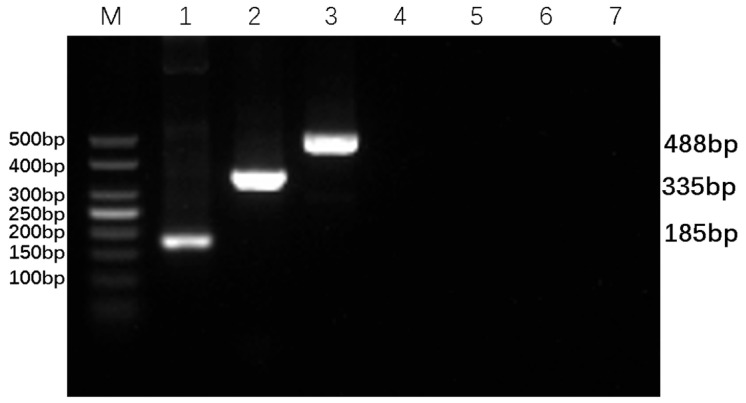
Detection of BVDV, BAstV, and BRV using the DPO-nanoPCR assay. Lane M contains a 50 bp DNA Ladder Marker for size verification. Lane 1 shows the amplification product of BVDV, lane 2 of BAstV, and lane 3 of BRV.

The optimized DPO-nanoPCR reaction system (50 µL) consisted of: 1 µL of each cDNA, 25 µL of 2× Taq PCR MasterMix (Biomed, Beijing, China), 0.4 µL of each forward and reverse BVDV-DPO primer, 0.3 µL of the BAstV-DPO primer, and 0.3 µL of the BRV-DPO primer, and 0.9 µL of GNPs (10 nM), with the volume adjusted to 50 µL using nuclease-free water. The reaction conditions were set as follows: initial denaturation at 95 °C for 5 min, followed by 35 cycles of 95 °C for 30 s, 55 °C for 30 s, and 70 °C for 40 s, and a final extension at 72 °C for 10 min. The amplicons were visualized using 2% agarose gels. As shown in Figure 5, the amplicons generated by the optimized DPO-nanoPCR were clear and specific, with a clean negative control, further validating the effectiveness of the optimized reaction system.

### 3.3. Sensitivity of the DPO-nanoPCR Assay

**Figure 6:**
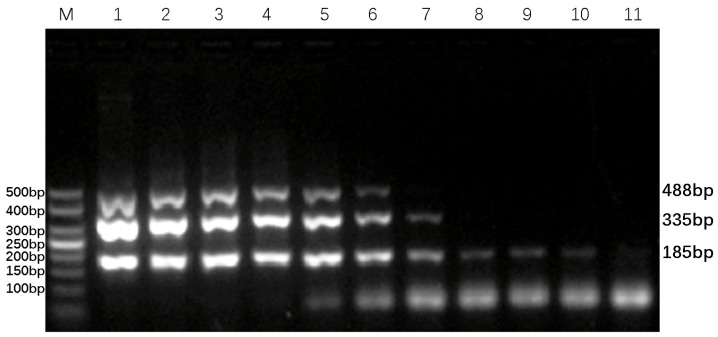
Sensitivity test of the DPO-nanoPCR. Lane M represents a 50 bp DNA Ladder Marker for size reference. Lanes 1–10 contain PCR products amplified using templates with concentrations of 10^−1^, 10^−2^, 10^−3^, 10^−4^, 10^−5^, 10^−6^, 10^−7^, 10^−8^, 10^−9^, and 10^−10^ ng/µL, respectively. Lane 11 is a negative control. Three technical replicates were performed with consistent results, and a representative gel image is shown.

The sensitivity of the established DPO-nanoPCR method was verified by diluting the BVDV, BAstV, and BRV positive plasmid standards (1 ng/µL) across a 10^−1^–10^−10^ dilution series. As shown in Figure 6, the lowest detection limits for BVDV, BAstV, and BRV were determined to be 1 × 10^−10^ ng/µL, 1 × 10^−7^ ng/µL, and 1 × 10^−6^ ng/µL, respectively, corresponding to copy numbers of 4.9 × 10^−1^ copies/µL, 2.72 × 10^2^ copies/µL, and 1.88 × 10^3^ copies/µL, respectively. The detection limits were consistently reproduced across all technical replicates. These results highlight the high sensitivity of the DPO-nanoPCR method in detecting low-abundance viral targets.

### 3.4. Specificity of the DPO-nanoPCR Assay

**Figure 7:**
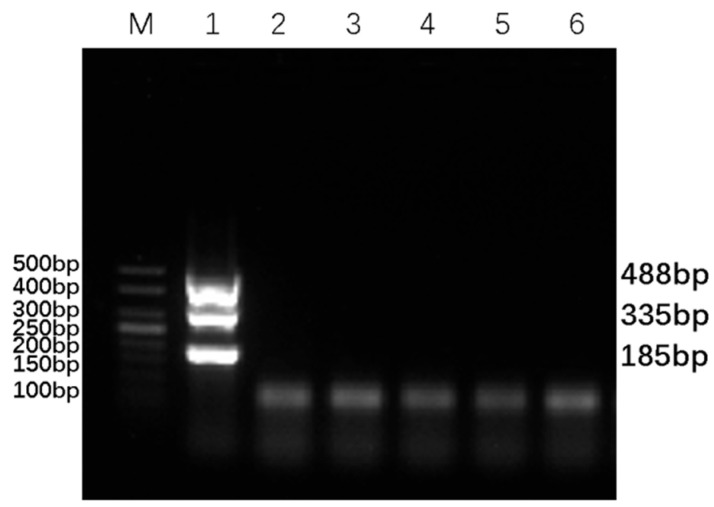
Specificity test of the DPO-nanoPCR. Lane M is a 50 bp DNA Ladder Marker for size comparison. Lane 1 contains a mixed template of BVDV clinical isolate cDNA, BAstV positive plasmid, and BRV positive plasmid, serving as a positive control. Lanes 2–6 represent the amplification results using cDNA or DNA templates from bovine coronavirus (BCoV), bovine parainfluenza virus 3 (BPIV3), infectious bovine rhinotracheitis virus (IBRV), atypical porcine pestivirus (APPV), and porcine epidemic diarrhea virus (PEDV), respectively. Three technical replicates were performed with consistent results, and a representative gel image is shown.

The specificity of the DPO-nanoPCR was analyzed by performing amplification reactions on DNA or cDNA samples from BCoV, BPIV3, IBRV, APPV, PEDV, as well as a mixed template of BVDV clinical isolate cDNA, BAstV-positive plasmid, and BRV-positive plasmid. As shown in Figure 7, only BVDV, BAstV, and BRV produced bright and clear specific bands, while no specific bands were observed for the other five viruses. These results strongly confirm that the DPO-nanoPCR method developed in this study has high specificity, ensuring accurate detection of the target viruses without cross—reactivity with other related pathogens.

### 3.5. Reproducibility of the DPO-nanoPCR Assay

**Figure 8:**
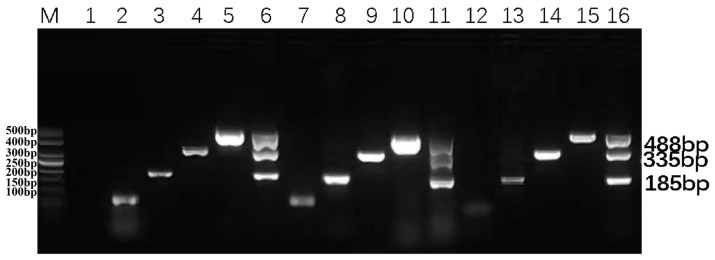
Repeatability test of the DPO-nanoPCR. Lane M shows a 50 bp DNA Ladder Marker for sizing the PCR products. Lane 1 is a blank control, while lanes 2, 7, and 12 are negative controls. Lanes 3–6 depict the amplification results using Tsingke 2× Premix Taq at 2—month intervals for BVDV, BAstV, BRV, and a mixture of BVDV + BAstV + BRV, respectively. Lanes 8–11 show the amplification results using Takara Bio 2× Premix Taq at 4-month intervals for the same set of viruses. Lanes 13–16 present the amplification results using Sangon 2× Premix Taq at 6-month intervals. Three technical replicates were performed with consistent results, and a representative gel image is shown.

To evaluate the reproducibility of the DPO-nanoPCR assay, single DPO-nanoPCR and multiplex DPO-nano PCR-specific amplifications were performed using the prepared positive plasmid standards as templates and 2× Premix Taq from different companies at 2-month intervals. As demonstrated in Figure 8, all amplifications successfully generated the expected bands, indicating that the established method has excellent reproducibility and stability, which is crucial for its reliable application in various experimental settings.

### 3.6. Detection of Clinical Samples

DPO-nanoPCR and conventional PCR were used to test 963 clinical samples. The results are summarized in Table 3. Forty-two (4.36%), six (0.62%), and nine samples (0.94%) were positive for BVDV, BAstV, and BRV, respectively, using conventional PCR. Fifty-three (5.50%), ten (1.04%), and nine samples (0.94%) were positive for BRV, BPV, and BVDV, respectively, using DPO-nanoPCR. Of all the clinical samples, 0.21% (2 out of 963) were positive for mixed infection by conventional PCR, and 1.04% (10 out of 963) were positive for mixed infection by DPO-nanoPCR (Table 3).

The positive results obtained by conventional PCR were highly consistent with those of DPO-nanoPCR (Table 4). For instance, 9 samples were positive for BRV by both methods, 10 samples were positive for BAstV by DPO-nanoPCR compared to 6 by conventional PCR, and 53 samples were positive for BVDV by DPO-nanoPCR versus 42 by conventional PCR. Notably, no samples showed negative results by DPO-nanoPCR but positive results by conventional PCR. Sequence analysis revealed a 100% similarity between the reference sequences of the target viruses and the DPO-nanoPCR amplicons. Collectively, these findings clearly demonstrate that the DPO-nanoPCR assay is more sensitive than the conventional PCR method, with compliance rates of 98.86%, 99.58%, and 100% for the detection of BVDV, BAstV, and BRV positive results in the total samples, respectively.

The chi-square test results for the detection rates of the two detection methods are shown in Table 4, with *p* values > 0.05 for both, proving that there is no significant difference in the detection rates between the two detection methods. The DPO-nanoPCR detection method is suitable for the detection of clinical samples.

## 4. Discussion

Virus-induced diarrhea in calves represents a severe disease that has caused substantial economic losses to the global dairy and beef cattle industries [30,31,32]. Developing rapid detection methods is fundamental for formulating effective prevention and control strategies. Among the diverse etiological agents, BVDV, BAstV, and BRV are recognized as primary enteric pathogens. Owing to their similar clinical manifestations and transmission routes, mixed infections are common [33]. Such mixed infections not only exacerbate the clinical symptoms of calf diarrhea but also complicate the accurate assessment of single-pathogen prevention and control measures, thereby escalating the economic losses in animal husbandry [34]. Consequently, there is an urgent need for a technical approach that can simultaneously detect these three pathogens, aiming to enhance diagnostic efficiency, conserve time and human resources, and mitigate losses in cattle farms.

In this study, multiplex DPO-nanoPCR detection methods was successfully developed for the simultaneous detection of BVDV, BAstV, and BRV. A key finding is the significantly higher sensitivity for BVDV and BAstV compared to conventional PCR. This superiority is attributed to the unique structure of DPO primers, which effectively prevents non-specific hybridization between primers and non-target sequences [19,35]. Such high specificity is paramount for identifying low-titer infections and occult cases in mixed infection scenarios. When compared with previous studies, Liu et al. (2021) established a quadruple real-time fluorescence quantitative RT-PCR assay with a limit of detection (LOD) of 10^3^ copies/µL for BAstV [36], whereas our study achieved a lower LOD of 2.72 × 10^2^ copies/µL, demonstrating enhanced sensitivity. Regarding other platforms, the triplex loop-mediated isothermal amplification-lateral flow dipstick (LAMP-LFD) method (Xu et al., 2024) reported LODs of 2.62 × 10^1^ copies/µL for BVDV and 2.43 × 10^1^ copies/µL for BRV [37], while the droplet digital PCR (ddPCR) assay by Chen et al. (2023) reached an LOD of 2.4 copies/µL for BRV [38]. Specifically, relative to the multiplex DPO-nanoPCR assay by Wang et al. (2019) (4.09 × 10^−1^ copies/µL for BVDV and 9.4 × 10^2^ copies/µL for BRV) [7], our assay yielded the LOD of 4.9 × 10^−1^ copies/µL for BVDV and 1.88 × 10^3^ copies/µL for BRV. These findings indicate that our assay’s sensitivity for BVDV is commensurate with Wang’s method and surpasses both LAMP-LFD and ddPCR, albeit displaying a marginal decrease in sensitivity for BRV. Such intra-assay variations in sensitivity may stem from differences in primer amplification efficiency and the quality of positive plasmid standards [39,40]. Although qPCR and ddPCR offer quantitative advantages, they typically require expensive equipment and specialized expertise, limiting their application in resource-limited field settings. Crucially, beyond the preliminary work of Wang et al. (2019) [7], this study provides a more rigorous validation of the system’s performance through large-scale on-site clinical trials and multi-condition repeated verification. These results underscore the method’s capacity to maintain diagnostic accuracy across diverse viral loads, facilitating its deployment in challenging field-level veterinary settings.

To evaluate the clinical relevance of the established approach, 963 fecal/anal swab and serum samples were analyzed using conventional PCR and DPO-nanoPCR. The BVDV detection rate observed in the present study (5.50%) was marginally higher than the infection range (0.34–4.90%) reported for yaks in Qinghai [41]. However, the prevalence of BAstV (1.04%) and BRV (0.94%) markedly diverged from the higher rates recorded in diarrheic cohorts in Ningxia [42]. This discrepancy is primarily attributable to our sampling strategy, which involved routine clinical screening of a population containing a high proportion of asymptomatic individuals. Notably, a co-infection rate of 1.04% was detected, with all BAstV-positive cases manifesting as mixed infections. This aligns with reports that BAstV predominantly circulates as a co-pathogen [43], suggesting a significant role in synergistic pathogenesis. Furthermore, the prevalence of the virus varies among cattle breeds, which is associated with their vaccination status and regional epidemic distribution.

The concordance rates between DPO-nanoPCR and conventional PCR for BVDV, BAstV, and BRV were 98.86%, 99.58%, and 100%, respectively. Conventional PCR performed adequately in detecting BRV, likely due to the high viral loads typically shed by diarrheic calves; however, DPO-nanoPCR demonstrated superior performance in identifying BVDV and BAstV. Unlike BRV, BVDV and BAstV infections often manifest with intermittent shedding or low-load characteristics, necessitating more sensitive diagnostic techniques. As previously established, PCR based on the DPO principle exhibits higher target sequence specificity than conventional methods [44,45]. Thus, the DPO-nanoPCR technology provides significant benefits in identifying low-abundance pathogens and diagnosing early infections, which is essential for interrupting transmission pathways, promptly isolating infected animals, and mitigating the spread of epidemic. Analytical sensitivity reflects the detection limit of our method using simulated samples, which can partially reflect diagnostic sensitivity. However, differences between the analytical and diagnostic sensitivity may exist due to the complexity of actual clinical conditions and various influencing factors [7].

While the assay does not allow for absolute quantification and showed slightly lower sensitivity for BRV—potentially due to the smaller target amplicons—its high sensitivity for BVDV and BAstV makes it superior for population-level screening. Although virus isolation was not performed, typical positive samples were verified via sequencing to ensure reliability. Future work will investigate potential prevalence variations across different cattle breeds and batch-to-batch variations of gold nanoparticles, so as to further refine this diagnostic tool for global veterinary applications.

## 5. Conclusions

Overall, the DPO-nanoPCR method demonstrated a higher detection rate in clinical samples compared to the conventional PCR method, with an overall consistency in detection results exceeding 98.86%. This method showcases excellent stability and reliability in accurately identifying BVDV, BAstV, and BRV, enhancing clinical diagnostic efficiency and reducing diagnostic time. It is particularly well-suited for the rapid screening and precise identification of low-copy-number and large-batch samples.

## Figures and Tables

**Table 1:** Primers used for conventional PCR and DPO-nanoPCR.

Primer Type	Gene	Name	Primer Sequences (5′→3′)	Product Size
Conventional	*5′-UTR*	BVDV-F	AGTACAGGGTAGTCGTCAG	179 bp
		BVDV-R	GTAGCAATACAGTGGACCTC	
DPO		DPO-BVDV-F	GCCCTGAGTACAGGGTAGTCGTCAGIIIIITGGTTCGAC	185 bp
		DPO-BVDV-R	GTAGCAATACAGTGGGCCTCTGCIIIIIAGCACCC	
Conventional	*ORF 1ab*	BAstV-F	AGCGCTCCTTAAACACATAA	354 bp
		BAstV-R	CATCTTGACGACTTGCTCTA	
DPO		DPO-BAstV-F	CGCGAGCGCTCCTTAAACACATAAAIIIIIAGACCTTAG	335 bp
		DPO-BAstV-R	AGTCATCAGGGACACACGGCGAIIIIITGATGAAAGACG	
Conventional	*VP6*	BRV-F	GGATCAGAAATTCAAGTCGC	514 bp
		BRV-R	GGTATCGCGTATTCTTGTCT	
DPO		DPO-BRV-F	TGGGTACGATGTGGCTCAATGCGIIIIIGGATCAGAGC	488 bp
		DPO-BRV-R	CACTTGCGTCGGCAAGCACTGAIIIIITTCACAAACT	

**Table 2:** Information of the recombinant plasmids.

Plasmid	Gene	Reference Strain Name	Genebank ID	Size of the Gene
pUC57-*5′-UTR*	BVDV *5′-UTR*	103	MW057258	211 bp
pUC57-*ORF 1ab*	BAstV *ORF 1ab*	HT4-TUR	MG957150	422 bp
pUC57-*VP6*	BRV *VP6*	ZAF/1162/2012	MW771109	1162 bp

**Table 3:** Detection of clinical samples by conventional PCR and DPO-nanoPCR.

Sample Source	Conventional PCR				DPO-nanoPCR			
	BVDV Positive Rate (%)	BAstV Positive Rate (%)	BRV Positive Rate (%)	Mixed Infection Positive Rate (%)	BVDV Positive Rate (%)	BAstV Positive Rate (%)	BRV Positive Rate (%)	Mixed Infection Positive Rate (%)
Meishan City	88.89 (8/9)	0 (0/9)	0 (0/9)	0 (0/9)	88.89 (8/9)	0 (0/9)	0 (0/9)	0 (0/9)
Guang’an City	20.00 (3/15)	0 (0/15)	0 (0/15)	0 (0/15)	20.00 (3/15)	0 (0/15)	0 (0/15)	0 (0/15)
Yibin City	0 (0/9)	66.67 (6/9)	0 (0/9)	0 (0/9)	0 (0/9)	66.67 (6/9)	0 (0/9)	0 (0/9)
Ya’an City	10.00 (3/30)	0 (0/30)	30.00 (9/30)	6.67 (2/30) ^1^	10.00 (10/30)	0 (0/30)	30.00 (9/30)	30.00 (9/30) ^1^
Zizhong City	17.39 (4/23)	0 (0/23)	0 (0/23)	0 (0/23)	17.39 (4/23)	0 (0/23)	0 (0/23)	0 (0/23)
Leshan City	45.45 (5/11)	0 (0/11)	0 (0/11)	0 (0/11)	45.45 (5/11)	36.36 (4/11)	0 (0/11)	9.09 (1/11) ^2^
Aba Prefecture	33.33 (4/12)	0 (0/12)	0 (0/12)	0 (0/12)	33.33 (4/12)	0 (0/12)	0 (0/12)	0 (0/12)
Ganzi Prefecture	1.76 (15/854)	0 (0/854)	0 (0/854)	0 (0/854)	2.22 (19/854)	0 (0/854)	0 (0/854)	0 (0/854)

^1^ Mixed infection of BVDV and BRV. ^2^ Mixed infection of BVDV and BAstV.

**Table 4:** Statistical analysis of the detection rates of different viruses in the total samples by conventional PCR and DPO-nanoPCR.

Method	Ratio of BVDV Positive Samples	Ratio of BAstV Positive Samples	Ratio of BRV Positive Samples
Conventional PCR	4.36% (42/963, 95%CI: 3.24–5.84%)	0.62% (6/963, 95%CI: 0.29–1.35%)	0.93% (9/963, 95%CI: 0.49–1.77%)
DPO-nano PCR	5.50% (53/963, 95%CI: 4.23–7.13%)	1.04% (10/963, 95%CI: 0.57–1.90%)	0.93% (9/963, 95%CI: 0.49–1.77%)
χ^2^	1.34	1.01	0
*p* value	0.25	0.32	1.00

## Data Availability

Data will be made available on request.

## Supplementary Materials

The following supporting information can be downloaded at: https://www.mdpi.com/article/10.3390/ani16060914/s1, Table S1: Clinical Sample Sources and Information in Sichuan Province.
