# Supplementary material for: A DPO-Enhanced Gold Nanoparticle-Assisted PCR Assay for Simultaneous Detection of Bovine Viral Diarrhea Virus, Astrovirus, and Rotavirus
Source: Animals (Basel). 2026 Mar 14;16(6):914. doi: 10.3390/ani16060914 (PMC13023237; doi:10.3390/ani16060914)
Supplement: Supplementary file 1 [file animals-16-00914-s001.zip › animals-4156844-supplementary.pdf]

Table S1. Clinical Sample Sources and Information in Sichuan Province.

**Area**	**Number**	**Type**	**Age**	**Breeds**	**Symptom**	**Time**
Meishan City	9	Anal swabs	Calves	Simmental	Diarrhea, High fever	2024.01
Guang'an City	15	Feces	Calves	Simmental	Diarrhea, High fever	2023.10
Yibin City	9	Feces	Calves	Simmental	Diarrhea	2023.02
Ya'an City	30	Feces	Calves	Simmental	Diarrhea	2023.09
Zizhong City	23	Feces	Mixed (Adult and Calves)	Simmental	Diarrhea	2023.12
Leshan City	11	Feces	Calves	Simmental	Diarrhea	2024.01
Aba Prefecture	12	Anal swabs	Calves	Yak	Diarrhea, Bloody stool	2024.01
Ganzi Prefecture Daofu County	442	Serums	Mixed (Adult and Calves)	Yak	-	2023.08
Ganzi Prefecture Hongyuan County	412	Anal swabs	Mixed (Adult and Calves)	Yak	-	2023.09
